# Editorial: Insights in clinical and translational physiology: 2023

**DOI:** 10.3389/fphys.2025.1508102

**Published:** 2025-01-21

**Authors:** Gaetano Santulli, Markus W. Hollmann, Christina M. Pabelick, Johannes J. Van Lieshout

**Affiliations:** ^1^ Department of Medicine (Division of Cardiology), Wilf Family Cardiovascular Research Institute, Einstein Institute for Aging Research, Albert Einstein College of Medicine, New York, NY, United States; ^2^ Department of Molecular Pharmacology, Einstein-Mount Sinai Diabetes Research Center (ES-DRC), Einstein Institute for Neuroimmunology and Inflammation (INI), Fleischer Institute for Diabetes and Metabolism (FIDAM), Albert Einstein College of Medicine, New York, NY, United States; ^3^ Department of Advanced Biomedical Sciences and International Translational Research and Medical Education (ITME) Consortium, Academic Research Unit, “Federico II” University, Naples, Italy; ^4^ Department of Anesthesiology, Academic Medical Centers, Division Amsterdam Cardiovascular Sciences, Amsterdam, Netherlands; ^5^ Department of Anesthesiology and Perioperative Medicine (Division of Pediatric Anesthesia), Mayo Clinic, Rochester, MN, United States; ^6^ Department of Internal Medicine, Laboratory for Clinical Cardiovascular Physiology, Academic Medical Centers, University of Amsterdam, Amsterdam, Netherlands; ^7^ Division of Physiology, Pharmacology and Neuroscience, Medical Research Council Versus Arthritis Centre for Musculoskeletal Ageing Research, Queen’s Medical Centre, School of Life Sciences, The Medical School, University of Nottingham Medical School, Nottingham, United Kingdom

**Keywords:** non-small-cell lung cancer (NLSCLC), circulating tumour DNA (ctDNA), mechanosensitive ion channels in lung cells, neural network, hemorrhage, sedentary behavior

Lung cancer is the main cause of cancer-related death, with non-small-cell lung cancer (NSCLC) as the common variant. Hitherto the paucity of tumour-related symptoms precludes early diagnosis in NSCLC (Franzi et al., 2023). The first paper of this topic by Xin et al.reports on advances in the diagnostic approach of patient with a NSCLC. The authors discuss the present state of art regarding the applicability and trustworthiness in early diagnosis and typing of circulating tumour DNA (ctDNA) as a liquid - rather than the usual solid - biopsy modality in patients with lung cancer.

In the second paper, Zheng et al. review role and potential impact of membrane proteins capable of responding to mechanical stress initiated by external mechanical stimuli with a focus on a variety of lung diseases. Many excitable tissues are sensitive to mechanical stimulation. A classical example of transmission of mechanical forces is the effect of intravascular pressure that is transduced into tension as sensed in the high- and low pressure areas by cardio- and pulmonary baroreceptors (Guharay and Sachs, 1984; Al-Timman et al., 1993; Roddie et al., 1957; Ledsome and Linden, 1964). It was found - for the first time in cultured chick muscle tissue - that membrane stress activates an ionic channel (Guharay and Sachs, 1984).

Zheng et al. discuss the effects of mechanosensitive ion channels in lung cells in a variety of lung diseases including pulmonary hypertension, asthma, acute respiratory distress syndrome, chronic obstructive lung disease and lung fibrosis.

Shenkin et al. wrote in their 1944 sentinel paper on detection of haemorrhage in humans: “*That hemorrhage can be diagnosed by a rapid pulse and a low blood pressure is a concept so widely accepted that it seems almost an impertinence to question it”* (Shenkin et al., 1944). This was subsequently verified in consecutive patients during resuscitation from haemorrhagic shock (Sander-Jensen et al., 1986). In general, providing volume treatment based on cardiovascular variables is heavily debated (Secher et al., 2011). Traditional patient monitoring in the emergency ward and the operating room includes heart rate and blood pressure but their use as predictors for incipient central hypovolemia is rather limited (van der Ster et al., 2021).

During World War II, the observation was repeatedly made that air raid victims in London City suffering from major blood loss presented with relative bradycardia rather than the expected tachycardia (Grant and Reeve, 1941). It is problematic that no single physiological variable responds exclusively to a reduced central blood volume (Secher and Van Lieshout, 2005). In the battle field, haemorrhage is a major cause of soldier death (Soller et al., 2012) and recently machine-learning algorithms for determination of blood volume status have been developed (van der Ster et al., 2021; Rickards et al., 2014; van der Ster et al., 2018). Against that background Jin et al. consider the potential for incorporating neural network-based AI technologies applied to created synthetic vital-sign data in hemorrhage detection and treatment.

Sedentary behavior has been defined as an immobile state of the body (e.g., sitting) resulting in energy expenditure close to the resting metabolic rate. In a study in 1084 Finnish women and 909 men aged 30–45 years, TV viewing was the type of sedentary behavior most consistently associated with adiposity markers in adults (Heinonen et al., 2013) and in a twin study the impact of physical activity on the circulating metabolome was verified (Kujala et al., 2013). In the fourth paper in this series Heinonen considers the clinical and physiological advances of modifying sedentary behavior. The data were gathered from a randomized controlled trial investigating the physiological and clinical benefits of reduced daily sitting in metabolic syndrome patients during a 6-month intervention. Not unexpectedly, replacing sitting by movement rather than by standing seems more beneficial for fitness and health.
